# Transfusion Thresholds, Quality of Life, and Current Approaches in Myelodysplastic Syndromes

**DOI:** 10.1155/2016/8494738

**Published:** 2016-04-19

**Authors:** Ioannis Koutsavlis

**Affiliations:** Haematology Department, NHS Lothian, Edinburgh EH4 2XU, UK

## Abstract

Hemoglobin thresholds and triggers for blood transfusions have changed over the years moving from a higher to a lower level. This review article summarizes the current evidence of transfusion thresholds in the hospitalized as well as in the outpatient setting and particularly in myelodysplasia. Fatigue is the main reported symptom in this group of patients and current clinical trials are looking for a more liberal approach of red cell transfusion and the effect on quality of life as opposed to the restrictive strategy used in the critical care setting. Practical considerations, the cost effectiveness of this strategy in addition to the possible complications, and the use of quality of life questionnaires have also been reviewed.

## 1. Introduction

There is ongoing research seeking the ideal hemoglobin threshold which would trigger blood transfusion in different clinical scenarios but the debate remains. The central concept for most studies has been the outcome of restrictive versus liberal transfusion strategies. Previous research has shown that in coronary artery bypass procedures and in patients undergoing major arterial reconstruction a lower hemoglobin threshold of 8 g/dL or 9 g/dL, respectively, does not adversely affect patient outcome [1–3]. Similarly, in orthopedic procedures liberal transfusion did not show any benefit and in some cases may increase the use of blood products [4–7]. The Canadian Critical Care Trials Group showed that a restrictive red blood cell (RBC) transfusion strategy appears to be safe for critically ill multiple-trauma patients (hemoglobin concentration 7 g/dL versus 100 g/dL) [8, 9]. The recent TRIGGER trial [10] evaluated the outcome of a restrictive (hemoglobin 8 g/dL) versus a liberal approach (hemoglobin 10 g/dL) in patients aged 18 years or older with new presentations of acute upper gastrointestinal bleeding. There was no significant difference in clinical outcomes; however, there was a nonsignificant reduction in RBC transfusion in the restrictive policy. Although more knowledge is acquired towards the management of acute conditions, there is limited research regarding hemoglobin targets in chronic anemia and bone marrow failure syndromes. Transfusion thresholds may differ between practitioners and there is no international consensus on this matter. In particular, patients with myelodysplasia appear to be a challenging group. Blood transfusion when anemia causes symptoms is a pragmatic approach but compromises quality of life and may even have a negative impact on overall survival.

## 2. Liberal versus Restrictive Transfusion: Systematic Reviews and Economic Impact

A systematic review from Carson et al. which included 19 trials with a total of 6264 patients showed that restrictive transfusion strategies did not appear to impact the rate of adverse events compared to liberal transfusion strategies (i.e., mortality, cardiac events, myocardial infarction, stroke, pneumonia, and thromboembolism). The researchers included only randomized controlled trials with a concurrent control group and surgical or medical patients, involving adults and/or children but not neonates. Eight studies took place within the context of surgery: cardiac, vascular, or orthopaedic. Five trials were in the context of acute blood loss and/or trauma, three trials involved patients in critical care units, and one trial involved leukemia patients undergoing chemotherapy or stem cell transplantation. Restrictive transfusion strategies were associated with a statistically significant reduction in hospital mortality (RR 0.77, 95% CI 0.62 to 0.95) but not 30-day mortality (RR 0.85, 95% CI 0.70 to 1.03). The use of restrictive transfusion strategies did not reduce functional recovery and hospital or intensive care length of stay [11]. Another very recent meta-analysis of 31 randomized trials totalling 9813 patients showed that restrictive compared with liberal transfusion strategies were not associated with risk of death, overall morbidity, or fatal or nonfatal myocardial infarction. This group included both single and multicentre randomized controlled trials. Population sizes ranged from 25 to 2016 and eight trials included more than 500 patients. The clinical settings of most of the trials were perioperative and acute blood loss (20 trials), critical care (eight trials), and trauma (two trials). One trial included patients with leukemia undergoing stem cell transplantation. Results were not affected by the inclusion of studies with unclear or high risk of bias. Using trial sequential analyses on mortality and myocardial infarction, the required information size was not reached, but a 15% relative risk reduction or increase in overall morbidity with restrictive transfusion strategies could be excluded. The authors concluded that, compared with liberal strategies, restrictive transfusion strategies were associated with a reduction in the number of red blood cell units transfused and number of patients being transfused, but mortality, overall morbidity, and myocardial infarction seemed to be unaltered. Restrictive transfusion is safe in most clinical settings and liberal transfusion strategies have not been shown to convey any benefit to patients [12]. From the above, it would appear that a restrictive strategy is at least noninferior or to some extent beneficial compared to a liberal approach and many centres are adopting this to their local guidelines.

The economic burden has also been evaluated in different studies. In a single institution study by Ejaz et al., with 942 patients undergoing major abdominal surgery who received a transfusion, 456 units (11.4%) were transfused using a liberal trigger. By adopting a restrictive trigger, total overall RBC transfusion costs may have been reduced by $100 320 to $346 560 during the 44-month study period or $27 360 to $94 516 per year for patients undergoing a pancreas, liver, or colorectal resection [13]. A US study in MDS patients shows that the 3-year mean cumulative Medicare cost for a transfused patient is nearly three times that for a nontransfused patient ($88,264 versus $29,519) [14]. Greenberg et al. [15] estimated the annual costs of potentially anemia-altering drugs used to treat MDS ranging from $26,000 to $95,000 depending on the specific therapy, against an estimated cost of RBC transfusions and iron chelation therapy of $41,412. This suggests that the economic impact of drug therapy needs to be weighed against the potential for improvement in transfusion requirement, clinical outcomes, and quality of life (QoL). Goss et al. [16] calculated the annual economic burden of best supportive care in 61 transfusion-dependent MDS patients, including costs for erythropoiesis-stimulating agents (ESAs), RBC and platelet transfusions, transfusion related laboratory tests, iron chelation, and costs related to disease complications and adverse events. The total annual cost per patient to the US healthcare payer in this setting was $54,940. However, a multicentre, parallel-group trial from 17 centres in the United Kingdom comparing restrictive versus liberal strategies after cardiac surgery showed that total costs did not differ significantly between the groups. Mean costs associated with red cell units were £287 in the restrictive-threshold group and £427 ($713) in the liberal-threshold group (*P* < 0.001). Other cost components and total mean costs up to 3 months after surgery were similar in the two groups (£10,636 in the restrictive-threshold group and £10,814 in the liberal-threshold group) [17]. It would be interesting to see the cost effectiveness of such a strategy in chronic anemia and patients with myelodysplasia.

However, the above systematic reviews are limited to patients following a surgical intervention and in the critical care unit but have not included chronic anemia or disorders of bone marrow failure.

## 3. Transfusion Thresholds and Clinical Trials in MDS

Transfusion guidelines have been published from different societies. As an example, the AABB (American Association of Blood Banks) recommends that transfusion, in hemodynamically stable patients with no active bleeding, is indicated almost always with Hb < 6 g/dL or not indicated when Hb > 10 g/dL except in exceptional circumstances. The lower threshold may vary between 6 g/dL and 8 g/dL but generally likely to be indicated when Hb < 7 g/dL [18]. The guidelines also emphasize that the decision to transfuse not only should be based on hemoglobin level but also should incorporate individual patient characteristics and symptoms. Clinical judgment is critical in the decision to transfuse; therefore, transfusing RBCs above or below the specified hemoglobin threshold may be dictated by the clinical context. Similarly, the decision not to transfuse RBCs to a patient with a hemoglobin concentration below the recommended thresholds is also a matter of clinical judgment [19]. Targets may differ, for example, in early severe sepsis and evidence of tissue hypoxia which may be 9-10 g/dL. The hemoglobin target in traumatic brain injury and/or cerebral ischemia could be 9 g/dL and in acute coronary syndrome 8-9 g/dL [20]. In relation to MDS patients, both the European Leukemia Network and BCSH guidelines advise that the objective of RBC transfusion therapy is to improve QoL and to avoid anemia-related symptoms and ischemic organ damage. The threshold hemoglobin concentration for transfusion will vary from patient to patient due to comorbidities, such as chronic pulmonary disease and heart failure; therefore no single recommendation for a transfusion trigger hemoglobin concentration can be made [21, 22].

A restrictive approach, as described in the above studies, has a consequence of a hemoglobin level as low as 7 g/dL and there may be some benefit in the acute setting and for the hospitalized patient, but in myelodysplasia and the chronically transfused patient, the outcome may be different. The negative implications of severe anaemia in MDS are well recognised. A retrospective study adopting a Cox proportional hazards regression model with time-dependent covariates found that hemoglobin levels lower than 9 g/dL in males and 8 g/dL in females were independently related to reduced overall survival and higher risks of nonleukemic death and cardiac death [23]. Moreover, there is a strong relationship between lower hemoglobin levels and worse cardiovascular outcomes, including cardiac remodeling, congestive heart failure, coronary artery disease, myocardial infarction, arrhythmia, heart valve disease, and cardiovascular mortality. Anemia is an independent predictor of cardiovascular disease outcomes in patients with MDS, beyond transfusion status and IPSS [24]. Therefore, it is common practice to keep Hb levels above 8-9 g/dL but evidence that retaining higher hemoglobin levels could improve overall survival is lacking. While this strategy prevents serious complications, including myocardial hypoxia and cardiac manifestations, it is at the cost of increased fatigue and overall reduced quality of life.

In the search for the ideal hemoglobin threshold, two feasibility studies are currently looking at transfusion thresholds and QoL in MDS. One from Canada is trying to compare the effect on QoL of a restrictive strategy (which is the current standard of care) with a liberal transfusion strategy in a randomized controlled trial of transfusion-dependent MDS outpatients. The experimental arm (liberal transfusion strategy) will maintain Hb level between 110 and 120 g/L. To achieve this, 2 units of RBCs are transfused when Hb level is <105 g/L and 1 unit of RBCs is transfused when Hb level is 105–110 g/L. The comparator arm (restrictive strategy) will have a threshold of Hb 85–100 g/L with 2 units of RBCs to be transfused when the Hb level is <80 g/L and 1 unit of RBCs when Hb level is 80–85 g/L [25]. Similarly, in the second trial (UK), patients are allocated to a restrictive Hb transfusion threshold (85–100 g/L) or a liberal threshold (110–125 g/L) policy with QoL as the primary endpoint. Before each transfusion patients will be asked to complete a short QoL questionnaire and another a week later. Each patient will be in the trial for 12 to 18 weeks [26].

**Figure 1:**
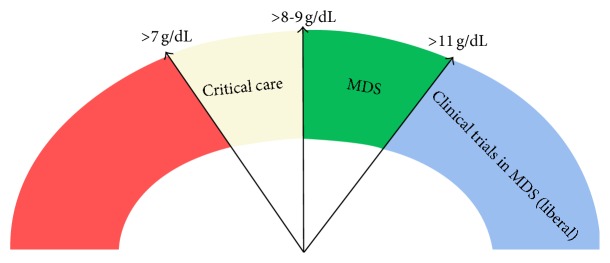
Transfusion thresholds. As a general rule in critical care a restrictive approach would be to transfuse red cells when Hb < 7 g/dL, whereas in myelodysplasia and likely other bone marrow failure syndromes more practitioners would transfuse when Hb is <8-9 g/dL. Current trials in MDS, looking for a liberal transfusion threshold and improved QoL, may set this target up to 11 g/dL.

Figure 1 summarizes a few transfusion thresholds in real practice.

## 4. Quality of Life in Myelodysplasia

As MDS is largely an incurable disease, the main focus over the years has been to improve QoL. Although quality of life encompasses the physical, mental, and social well-being, most of the studies have addressed this issue from the clinical perspective only. Fatigue is the most prevalent reported symptom in MDS [27] improved by correction of the anaemia. Therapeutic interventions could include growth factors and erythropoietin or simply red cell transfusion which aim to improve hemoglobin levels and eliminate the relevant symptoms of anaemia. A small study by Nilsson-Ehle et al. [28] assessed QoL, response rate, and physical function in elderly anemic MDS patients treated to a target Hb level of >120 g/L. Thirty-six elderly patients with low- and intermediate-1 risk MDS received darbepoetin (DA) 300 *μ*g/wk, with the addition of G-CSF if there is no response. Eighteen patients reached the target Hb level according to protocol. QoL scores for fatigue, dyspnoea, constipation, and physical, role, and social functioning improved significantly during study, in both transfused and untransfused patients. Similar results were obtained in the study of Oliva et al., where patients were treated with darbepoetin alpha (DPO). 41 patients received DPO 150 mg weekly for 24 weeks with the dose increasing to 300 mg weekly in nonresponsive patients. During treatment, 10/17 (59%) transfusion-dependent (TD) and 13/23 (56%) transfusion-free (TF) patients responded. In TF patients, Hb increased from 9.2 + 0.9 g/dL to 10.3 + 1.4 g/dL by 24 weeks. The mean response duration was 22 weeks in TF patients compared with 15.1 weeks in TD patients. Response to treatment was again associated with increases in QoL. The other important aspect of this study was the biological benefit of the ESAs with the reduction of both CD34+ cells and of their apoptotic compartment which seems to confirm previous in vitro data and indicates that this effect is one of the most important mechanisms of action of ESAs in counteracting the ineffective hematopoiesis of MDS. Furthermore, this may be one of the mechanisms through which responsive patients obtain a survival advantage [29].

QoL is so much individualized and could differ significantly among patients, making an objective assessment more difficult. The use of questionnaires may have helped, but most QoL analyses for MDS have used generic or cancer-specific instruments. At least 9 quantitative instruments have been used in studies of myelodysplastic syndromes with 9 to 38 items each [30]. The European Organization for Research and Treatment of Cancer QLQ-C30 (EORTC QLQ-C30) has been used in most studies with a global QoL measured [31]. The Functional Assessment of Chronic Illness Therapy (FACIT) measurement system is a collection of QOL questionnaires targeted to the management of chronic illness. This group of researchers has modified a backbone questionnaire to match different malignant and chronic disorders and one for patients with anaemia/fatigue also exists [32]. The brief fatigue inventory, developed by the MD Anderson Cancer Centre group, targets the most common symptom encountered in this group of patients and with only 4 questions (9 items), it is practical and easy to use [33]. It would seem reasonable to focus on this symptom not only due to its frequency but also because self-reported fatigue severity predicts overall survival beyond gold-standard prognostic indices in patients with higher risk myelodysplastic syndromes [34].

Involvement in treatment decisions by MDS patients can also be associated with their QoL, and it is therefore crucial to rely on solid measures. Dr. Abel and his colleagues developed a disease-specific measure of quality of life for patients with myelodysplastic syndromes. The most significant part of this effort was to host several focus groups for MDS patients, caregivers, and healthcare providers and then pilot the resulting measure—the Quality of Life in Myelodysplasia Scale or QUALMS—individually with a new group of patients. The purpose of the focus groups was to hear directly from MDS patients and their health care providers (inpatient and outpatient nurses, physician assistants, social workers, and physicians) regarding the factors that are most important to the quality of life of MDS patients and how we could best capture those factors in a questionnaire. Dr. Abel's group then created a 38-item questionnaire using data from the focus groups and have piloted and revised the questionnaire with the input of 20 new patients with MDS. Moving forward, they plan to validate the measure for use in clinical trials by administering it to a much larger group of patients from additional institutions, specifically measuring how responses on the QUALMS-1 change with changes in therapy and disease status. The domain with the highest ranking was again fatigue [35].

Since MDS is a dynamic condition, sometimes necessitating a more aggressive transfusion programme, or transform to acute myeloid leukaemia, QoL questionnaires may need to be repeated many times during this process.

## 5. Long Term Red Cell Transfusion: Improved QoL or Increased Transfusion Related Complications? Practical Considerations (Table 1)

The more the number of red cells transfused, the better the hemoglobin level and likely improved QoL. However, multiple transfusions and the serious ramifications, including iron overload, alloimmunization, allergic reactions, and infections, pose a huge burden not only on the patient but also on the health budget. Data from the European LeukemiaNet (ELN) MDS registry revealed that, amongst 1000 MDS patients with low- and intermediate-1 risk score, the mortality rate in transfusion-independent and transfusion-dependent subjects at 18 months of follow-up was 5% and 21%, respectively (*P* < 0.0001). Transfusion burden is the most important prognostic factor for survival in patients with more than 20 units transfused compared to nontransfused patients. An increased risk was also apparent for moderately transfused patients (<20 units) so direct toxicity of transfusions (toxic iron radicals?), even at a relatively low iron load, might have an important adverse impact on survival [36].

In the annual 2014 UK SHOT (Serious Hazards of Transfusion) report, there were 1681 transfusion related events with 389 acute and hemolytic transfusion reactions and 151 cases of alloimmunization. It is worth noticing that the morbidity and mortality data showed 15 deaths in which transfusion reaction was causal or contributory and 169 cases in which major morbidity probably or definitely attributed directly to transfusion reaction [37]. Additionally, there is an increased incidence of alloimmunization in MDS patients with current evidence suggesting an estimated rate somewhat within 15–30% [38, 39]. With most common antigens found from Rhesus and Kelly groups, extended molecular matching for Rh and K reduces red blood cell alloimmunization, in one study up to 65% [38, 40].

Iron overload is a well recognised consequence of long term transfusion and is associated with an increased risk of morbidity and mortality [41–43]. de Swart et al. evaluated the overall survival based on ferritin status in three different groups of patients according to their ferritin levels: group 1: <300 *μ*g/L; group 2: >300 and <1000 *μ*g/L; and group 3: >1000 *μ*g/L. The mortality rate according to serum ferritin at registration in these groups was 6%, 14%, and 23%, respectively, with HRs 1.80 (95% CI 1.06–3.03) and 3.21 (95% CI 1.66–6.20), including adjustment for transfusion status and number of transfusions [36]. Unfortunately, there is no robust evidence of how to best manage these patients, as all the studies are retrospective and methodologically limited [44]. Despite the above, there is almost national and international consensus for iron chelation therapy in selected MDS patients [45]. The BCSH guidelines suggest that consideration may be given to chelation therapy for patients with a very good prognosis, specifically patients with WHO RA, RARS, and isolated del(5q). Triggers may include more than 20 units of red cells transfused, serum ferritin >1000 *μ*g/L, in patients for whom continuing red cell transfusion is predicted [21]. This is supported by the Düsseldorf MDS registry study in which matched pair analysis of 188 patients showed that median overall survival was significantly better in chronically transfused but iron-chelated patients (75 months) than in matched patients without chelation therapy (49 months). In addition, patients with ongoing transfusion need whose serum ferritin decreased over time had a better survival as compared to patients whose ferritin levels increased despite iron chelation [46]. However, in advanced disease and patients with intermediate-2 or high IPSS score and median survival of 1.2 and 0.4 years, respectively, iron overload may not play a prevalent role as it will not influence overall survival. Sensitive magnetic resonance imaging (MRI) techniques have shown that cardiac iron overload may occur only at a very late stage of a patient's transfusion history [47–49]. Therefore, in this group of patients, iron chelation strategies are not usually adopted as part of a treatment approach.

To alleviate anemia-related symptoms and improve QoL, a holistic approach should be incorporated in the management of MDS patients. From the clinical perspective, it has been shown that large fluctuations in the hemoglobin levels may affect QoL. Lowering the amplitude of Hb fluctuations could improve QoL in elderly MDS patients. One approach could be to transfuse these patients more frequently but with fewer units of red cells [50]. Extended matching for Rhesus and Kelly blood groups may reduce the incidence of alloimmunization. The use of GCSF and/or erythropoietin should be recommended in patients with high probability of positive response as indicated in international guidelines. Iron chelation should be offered in certain group of patients. In theory, patients with preexisting liver and/or cardiac disease are more prone to end organ damage from iron overload. Consideration for chelation therapy should be given to these patients even with high risk IPSS. However, only randomized controlled trials can answer this hypothesis. Practitioners should also focus on the emotional and not only the physical needs of these patients. Appointment time constraints should be minimised when possible and patients always actively engage with discussions on their management. To this end, QoL questionnaires may significantly help with treatment decisions and should be repeated in different time points.

Although attending hospital for blood product support may offer many other advantages, from both the clinical point of view and the interaction of patients with the nursing staff, transport can be a burden for the frailer patient. The concept of blood transfusion at home dates back to the 1970s and may appear as an attractive solution in this setting. Protocols have been developed for the selection of patients and with a multidisciplinary approach, home transfusions can be a positive and vital part of a patient's care plan, which can increase their quality of life and reduce hospital burden [51]. However, despite the advantages, there are risks to home transfusion that must be carefully considered, the most urgent of which is the lack of immediate medical assistance in the event of an adverse transfusion related reaction [52]. Practitioners and health care organizations must carefully assess each patient's appropriateness for receiving this specialized care in their own home, considering their quality of life and the availability of quality care and ensuring that the benefits of the treatment outweigh the risks. A study by Niscola et al. [53] looked at the feasibility of transfusing blood at home in MDS patients. Home transfusion was initiated with the goal of providing more convenient supportive care to patients who have difficulty in travelling back and forth from the clinic. The major goal of the program was to improve the quality of life for the patient and caregiver. The patients were very old (median age: 85) and transfusion-dependent. These patients had exhausted their treatment options and had compromised functional status or social situations that made it impossible to regularly travel to the clinic or hospital. In total, 211 patients were enrolled in the program over a 5-year period. The median number of packed red blood cell units given was 38 per patient (range: 1–162) for a total of 7766 units that were given in 4980 home transfusions. The complication rate was low: there were 12 adverse events in 4980 home transfusions. All complications were managed in the home and there were no hospital admissions caused by home transfusions. This study demonstrated that home transfusion was feasible and could be done in a population of sick elderly patients with few complications. The impact of this study, however, is diminished by the inability of the study to measure benefits, such as quality of life, patient function, caregiver freedom, and assessing the cost effectiveness [54].

## 6. Conclusion

The lack of clinical trials for transfusion thresholds in chronic anemia and bone marrow failure syndromes has resulted in exclusion of this group of patients from national and international guidelines. Although a pragmatic approach would be to treat symptomatic anemia, this could be at the cost of quality of life. To complicate matters, patients with myelodysplasia are usually elderly with a background of cardiac and/or respiratory comorbidities. In the era of new drug development and personalised medicine, transfusion thresholds in myelodysplasia may be a myth and an individualized approach should be adopted. Patients should be at the centre of treatment decisions and more high quality clinical trials are needed to answer this question.

## Figures and Tables

**Table 1:** Practical considerations in myelodysplasia.

Improving quality of life	Enrol patients in clinical trials with liberal transfusion thresholds where possible Avoid large fluctuations in hemoglobin levels Adopt a holistic approach including the psychological and social needs of the patients Consider home transfusion strategies for the frailer patient Use validated QoL questionnaires in different time points

Minimizing transfusion related complications	Use growth factors and erythropoietin stimulating agents as per international guidelines Enrol patients in clinical trials with iron chelation therapies Consider extended matching for the Rhesus and Kelly blood groups to minimize the risk of alloimmunization
